# The Application of Iron Nanoparticles Green‐Synthesized by *Coptis chinensis* Leaf Aqueous Extract in Reducing the TNF‐α and IL1‐β Inflammatory Cytokines in the Rat Periodontal Model

**DOI:** 10.1002/fsn3.71492

**Published:** 2026-01-30

**Authors:** Jialing Wu, Xinjie Gao, Ruiqi Li

**Affiliations:** ^1^ Department of Prosthodontics, Stomatological Hospital, School of Stomatology Southern Medical University Guangzhou China; ^2^ Department of Periodontology The Maternal and Children Health Care Hospital (Huzhong Hospital) of Huadu Guangzhou China

**Keywords:** *Coptis chinensis*, IL1‐β, iron nanoparticles, periodontitis, TNF‐α

## Abstract

The study purpose was to examine how the green‐synthesized iron nanoparticles (FeNPs) made from *Coptis chinensis* extract affected the TNF‐α and IL1‐β gingival levels in the rat periodontal model. The extract from *C. chinensis* was utilized as a green reducing agent and a great stabilizer for the Fe NPs that were produced. The as‐synthesized Fe NPs were physicochemically characterized using FT‐IR, UV–Vis, EDX, XRD, and FE‐SEM. Male rats were given 0–3 ligatures around the neck of their right mandibular first tooth to develop inflammatory periodontitis. In the positive control group, indomethacin (5 mg/kg) was administered daily. As a pre/post treatment, Fe NPs (0.1 mg/kg) were directly injected into the gum tissue after being dissolved in dimethyl sulfoxide. ELISA was used to assess the TNF‐α and IL1‐β gingival levels. The Fe‐O bond is identified in the FT‐IR as the vibration band at 568 cm^−1^. The UV–Vis data indicate that FeNPs are linked to the band at 294 nm. The peaks in the collected data at 6.44 keV for FeKα, 7.13 keV for FeLβ, and 0.71 keV for FeLα proved the presence of iron in the EDX. The signals are indexed as (311), (400), and (440) planes with 2*θ* values of 38.3, 44.3, and 64.5. The findings showed that the rat periodontal model's gum tissue could produce less TNF‐α and IL1‐β when Fe NPs were administered (*p* ≤ 0.01). Additionally, the study's findings showed that gingival tissue in a periodontitis model had higher levels of IL1‐β and TNF‐α than the control group (*p* ≤ 0.01). The anti‐inflammatory efficacies of indomethacin and Fe NPs did not differ significantly. Because *C. chinensis* extract directly inhibits pro‐inflammatory cytokines, it can be used to reduce inflammation in a rat periodontal model both before and after treatment with green‐synthesized FeNPs.

## Graphical abstract

Treatment of periodontitis by the iron nanoparticles.

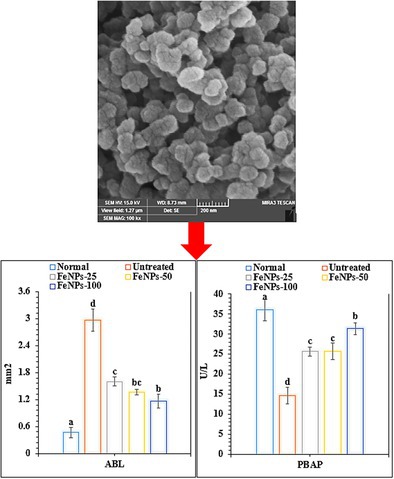

## 1 Introduction

The tissues that support the periodontium are impacted by the inflammatory illness known as periodontitis. It is brought on by the gums' immune response to plaque, which destroys the tissues that hold the gums together. One of the most well‐known dental conditions in the world, periodontitis plays a significant role in adult tooth loss (Jose et al. 2011; Pasupuleti et al. 2023). For patients with chronic periodontitis, root planing and scaling are still essential. Nevertheless, exclusive reliance on mechanical therapies such as extractions, periodontal surgery, and root planing is insufficient to prevent the progression of periodontitis (Jose et al. 2011; Pasupuleti et al. 2023; Gościniak et al. 2021). Gram‐negative anaerobic microorganisms, such as *Treponema species*, *Streptococcus intermedius* , *Campylobacter rectus* , *Fusobacterium nucleatum* , *Bacteroids forsythias*, *Prevotella intermedia* , *Porhyromonas gingivalis*, and *Aggregatibacter actinomycetemcomitans* , are the periodontal pathogens majority linked to periodontal and gingival diseases (Jose et al. 2011; Pasupuleti et al. 2023). When the mix of bacteria changes from Gram‐positive aerobic to Gram‐negative anaerobic germs, gingival and periodontal disease develops. According to epidemiologic research, antimicrobial therapy and nonsurgical therapy as an adjuvant aid in lowering the overall bacterial burden (Gościniak et al. 2021; Rani et al. 2022; More et al. 2008). Recently, both dental professionals and patients have become more interested in using herbal products and medications to treat dental caries, periodontitis, and gingivitis. Herbal materials, herbs, products, and preparations with plant parts or other plant components as active ingredients are all considered herbal remedies (Malhotra et al. 2011). To prevent inflammatory periodontal diseases, phytochemicals—bioactive plant compounds with wound‐healing, antibacterial, and antimicrobial efficacies—are the main components of therapeutic plants, including flavonoids, terpenoids, cardiac glycosides, saponins, alkaloids, tannins, steroids, anthraquinones, phlobatannins, and reducing sugars (Malhotra et al. 2011; Liu et al. 2019).

A traditional Chinese plant with a variety of medicinal uses is *Coptis chinensis*, or huanglian in Chinese. Berberine, its primary active ingredient, has anti‐diabetic, anti‐inflammatory, and antibacterial qualities (Li et al. 2023; Liu et al. 2015; Lin et al. 2024; Zheng et al. 2024; Yang et al. 2021). It has long been used to treat digestive problems like ulcers and inflammation of the stomach (Liu et al. 2021; Zhou et al. 2023). Its potential to cure depression, arthritis, and even some neurodegenerative illnesses is also being investigated in contemporary research (Li et al. 2023; Yang et al. 2021). Traditionally, *C. chinensis* has been used to treat digestive tract problems, duodenal ulcers, and stomach inflammation. It is frequently combined with other herbs to treat diseases of the digestive system, diarrhea, and enteritis (Li et al. 2023; Yang et al. 2021; Zhou et al. 2023). According to research, *C. chinensis* may have neuroprotective qualities that could aid in the treatment of neurological illnesses like Parkinson's, depression, and memory loss (Lin et al. 2024). It can improve autophagy, a cellular mechanism that eliminates faulty or damaged components, and decrease α‐synuclein aggregation, a major contributing factor to Parkinson's disease (Li et al. 2023; Yang et al. 2021). One important ingredient, berberine, has antibacterial and anti‐inflammatory qualities. It works well against a variety of bacteria and can lessen inflammation brought on by diseases like arthritis (Li et al. 2023). The main components of *C. chinensis*, jatrorrhizine hydrochloride, magnoflorine, jatrorrhizine, coptisine, palmatine, and berberine, play a role in treating the inflammatory diseases such as periodontitis by regulating PI3K/AKT/mTOR and Wnt1/β‐catenin signaling pathways (Li et al. 2023; Yang et al. 2021).

Periodontitis can be treated with green‐synthesized nanoparticles made from medicinal plants because they have unique therapeutic qualities, such as remarkable antioxidant, anti‐inflammatory, antibacterial, and immunomodulatory efficacies (Wang et al. 2024; Nasiri et al. 2023). However, a number of issues, including toxic buildup in cells, poor in vitro‐in vivo correlation, and poor animal‐to‐human transmissibility, restrict the therapeutic translation of nanoparticles (Wang et al. 2024; Nasiri et al. 2023; Mlachkova et al. 2025; Tong et al. 2023). With benefits like targeted medication delivery and improved antibacterial and regenerative qualities, nanoparticles are showing promise as a novel treatment for periodontitis. To enhance the treatment of periodontal disease, they can be added to a variety of drug delivery methods, such as hydrogels and nanofibers (Wang et al. 2024). FeNPs are being explored as experimental tools against periodontitis, mainly as antimicrobial, biofilm‐disrupting, and pro‐regenerative agents, but they are not yet part of standard clinical periodontal therapy in humans. Superparamagnetic FeONPs and Fe_3_O_4_‐based particles are used to physically penetrate and disrupt dense periodontal biofilms, often while carrying antibiotics such as minocycline, which enhances killing of plaque‐associated pathogens in vitro and in animal models (Tong et al. 2023). Magnetic guidance and local magnetic fields allow Fe_3_O_4_ nanoparticles to accumulate at periodontal pockets, improving localization and enabling stronger anti‐biofilm effects while maintaining acceptable biocompatibility in preclinical studies (Tong et al. 2023).

According to the above information, the recent FeNPs were green‐synthesized by the *C. chinensis* aqueous extract for therapeutic aims. Due to the *C. chinensis* having significant anti‐inflammatory effects against pro‐inflammatory cytokines, it seems that the synergistic effects of *C. chinensis* extract and iron reaction in green formulation make for higher anti‐inflammatory effects. This study demonstrated the strong anti‐inflammatory properties of green‐synthesized FeNPs by *C. chinensis* extract in an in vivo setting for the first time. We employed UV–Vis, FE‐SEM, XRD, FT‐IR, and EDX to determine the characteristics of the FeNPs that were created when an iron salt solution and aqueous *C. chinensis* extract reacted.

## 2 Experimental

### 2.1 Materials

All of the chemicals needed were bought from Sigma‐Aldrich and Merck. All of the compounds were utilized without purification.

### 2.2 Preparation of *Coptis chinensis* Leaf Extract

*Coptis chinensis* leaves were bought from a local market and left to dry for 10 days in the shade. After that, they were broken into tiny bits and sieved through 60 mesh sizes to create a fine powder. A volume of 100 mL of double‐distilled water was created by shaking 5 g of powder to create the root aqueous extract. After 30 min of heating at 60°C in a water bath, the extract was collected by filtering it using Whatman filter paper.

### 2.3 Green Formulation of FeNPs

To biosynthesize FeNPs, a solution containing 2 × 10^−2^ M FeCl_3_ was mixed 1:1 with the aqueous extract of *C. chinensis*. The dark brown that was seen suggested the creation of FeNPs. The reduced solution was centrifuged for 10 min at 10,000 rpm. Once centrifugation was complete, the supernatant was removed and discarded. To get rid of any contaminants, the pellets were cleansed three times using deionized water.

### 2.4 Chemical Characterization of FeNPs

FeNPs' distinctive surface plasmon resonance was measured with a Biospec‐1601 UV–vis spectrophotometer manufactured by Shimadzu Corporation in Kyoto, Japan. To do this, about 2 mL of the sample was taken and put into Falcon tubes with the proper labels so that the maximum absorption properties linked to the nanoparticle production could be examined using UV–Vis spectroscopy. At wavelengths between 200 and 800 nm, the nanoparticles were scanned. An energy dispersive spectrum (EDS) analysis and a FE‐SEM, Apreo Lo Vac, FEI, USA, were used to assess the elemental composition and surface topography of the FeNPs, respectively. High‐magnification pictures were taken using the FE‐SEM after the lyophilized powder of FeNPs was evenly modified with AuPd for 30 min at a width of 10 mm. Spectrophotometer of Cary 50 from 200 to 800 nm, Shimadzu 8400S FT‐IR Instrument at 400–4000 cm^−1^. A Bruker (Germany) diffractometer was used to perform XRD measurements at room temperature. Monochromatic X‐ray radiation (*λ* = 1.5406 Å) produced at 40 kV and 45 mA was used in the analysis. The 2*θ* range of 5° to 100° was used to record the diffraction patterns.

### 2.5 Periodontitis Experimental Model

All animal experiments were carried out according to the instructions of the Research Ethics Committee of the Ministry of Health and Medical Education on April 17, 2006, and are in accordance with the Helsinki Protocol (Helsinki, Finland, 1975). In this experimental study, 50 Wistar rats (*n* = 10) weighing between 240 and 260 g as adult males were purchased from the Animal Lab of Southern Medical University, S366 Jiangnan Boulevard, Haizhu District, Guangzhou, Guangdong, 510280, China; they were kept in a room at 20°C to 24°C, had unrestricted access to water and food, and all rats were carried out in strict compliance with the ethical standards for handling laboratory animals. A pelleted rodent diet, also referred to as lab blocks or rodent chow, was frequently given to laboratory rats. It is marketed as a commercial product and is designed to meet all of the nutritional requirements of rats. It was made by the LabDiet company in St. Louis, Missouri, USA. The sample size was calculated according to the following formula:

$$\text{Sample size}=2\;{\mathrm{SD}}^{2}\;{\left(Z^{\alpha /2}+\mathrm{Z\beta}\right)}^{2}/d^{2}$$

where standard deviation taken from a pilot study, *Z* ^*α*/2^ = *Z*_0.05/2_ = *Z*_0.025_ = 1.96 (from *Z* table) at type 1 error of 5%, *Zβ* = *Z*_0.20_ = 0.842 (from *Z* table) at 80% power, *d* = effect size = difference between mean values.

Hence, now the formula will be

$$\text{Sample size}=2\;{\mathrm{SD}}^{2}\;{\left(1.96+0.842\right)}^{2}/d^{2}$$

Five groups of ten rats each were created as follows:

1. Control group: No periodontitis induction was performed on this group; normal saline (2 mL per day) was the only post‐treatment administered to the rats of this group.
2. Untreated group: Periodontitis induction was done; normal saline (2 mL per day) was the only post‐treatment administered to the rats of this group.
3. Groups treated I: 2 mL per day FeNPs (25 μg/kg) were given post‐treatment to the rats of this group.
4. Groups treated II: 2 mL per day FeNPs (50 μg/kg) were given post‐treatment to the rats of this group.
5. Groups treated III: 2 mL per day FeNPs (100 μg/kg) were given post‐treatment to the rats of this group.

At the beginning of periodontitis induction, general anesthesia was achieved through intraperitoneal injection of ketamine hydrochloride (50 mg/kg) and xylazine hydrochloride (5 mg/kg). Inflammatory periodontitis was induced using 0–3 ligatures around the crown of the right first maxillary molar in rats, and to accumulate plaque in that area, the ligature was pressed into the sulcus. The ligatures were studied before the sacrifice. Left mandibular first molar was considered a control for each group. After induction of periodontitis using 0–3 suture around the crown of the right first maxillary molar ligatures were left in place for 7 days. In order to inject the normal saline and FeNPs separately, rats were anesthetized slightly with CO_2_. Then, normal saline and FeNPs, separately with a volume of 30 μL was injected into the rats gum tissue directly by Hamilton syringe (Sun and Yu 2024).

The commonly used immunological test, ELISA, was utilized to measure the levels of antioxidant enzymes and inflammatory cytokines (TNF‐α, IL1‐β, CINC‐1, and IL‐10) following the injection of FeNPs directly as a pre/post‐treatment into the gum tissue. Alveolar bone loss was measured in accordance with the previous study (Sun and Yu 2024). After the maxillae were fixed and demineralized, the slides were stained with hematoxylin and eosin for histological examination. To determine the mRNA expression of the RANK, TNF‐α, iNOS, and IL1‐β genes, qRT‐PCR analysis was carried out in accordance with the established protocol (Sun and Yu 2024).

### 2.6 Statistical Analysis

In order to ensure that every consecutive time, dilution, and ingredient had at least three repetitions, the study was conducted multiple times. To guarantee accuracy, the experiment was conducted at least three times. The results in this study were evaluated using the SPSS‐22 software and its tests, including one‐way ANOVA, and the post‐test (*p* < 0.05) was conducted using Duncan's method.

## 3 Results and Discussion

Based on a prior investigation, it was found that the plant extract has been observed to serve a dual function of both capping and reducing in the creation of metallic nanoparticles when utilizing green mediation methods. Within this particular research, the phytochemical compounds extracted from *C. chinensis* were noted to bind with Fe^3+^ leading to the aggregation of larger particles through processes such as coalescence or Oswald ripening. Additionally, the secondary metabolites from the plant were identified to be responsible for the iron ions, subsequently facilitating particle growth via aggregation. The size of the particles was noted to be affected by the quantity of Fe salt utilized and the biomolecules' presence inherent in the *C. chinensis* extract (Ma et al. 2023; Rezaee et al. 2022; Shi et al. 2021; Kgosiemang et al. 2023; Ahmadi et al. 2021).

Fe NPs were created by combining the *C. chinensis* with a pure FeCl_3_.6H_2_O solution, and after 24 h, the color of the resulting colloidal solution drastically altered to black. The reduction of Fe ions to FeNPs is known to cause color change, as reported by Ma et al. (2023), Rezaee et al. (2022), and Shi et al. (2021). Similar color shifts to black have also been observed by Kgosiemang et al. (2023) and Ahmadi et al. (2021). Based on the study of Namvar et al. (2014), the color change is associated with the Fe ions reduction to Fe0 as well as the SPR vibrations. Additionally, the previous study hypothesized that both SPR and electron oscillation are essential elements determining these color fluctuations (Li and Su 2025).

**FIGURE 1:**
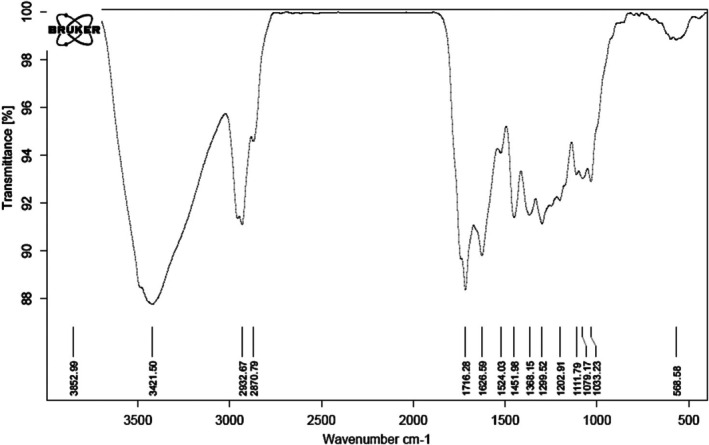
FT‐IR analysis of FeNPs@*Coptis chinensis* in the wavenumber of 400–4000 cm^−1^.

Green production of nanoparticles may be qualitatively investigated using the FT‐IR spectroscopy method. Metal‐oxygen connections are linked to the vibration bands in the wavenumber range below 700 cm^−1^. The organic functional groups present in the *C. chinensis* extract that are attached to the metallic nanoparticles surface are represented by the additional peaks. The FT‐IR spectra of FeNPs@*Coptis chinensis* are shown in Figure 1. The Fe—O bond is attributed to the vibration band at 568 cm^−1^, which is comparable to the previous studies (Shi et al. 2021; Kgosiemang et al. 2023; Ahmadi et al. 2021). Secondary metabolites of terpenoid, flavonoid, and phenolic compounds, which are major ingredients in *C. chinensis*, have bonds of C—O, C=C, C=O, C—H, and O—H, which may be represented by the vibration bands at the other regions, such as 1033, 1299 to 1716, 2870, and 3421 cm^−1^. The aforementioned connection is supported by the similarity between the plant and nanoparticle spectra.

**FIGURE 2:**
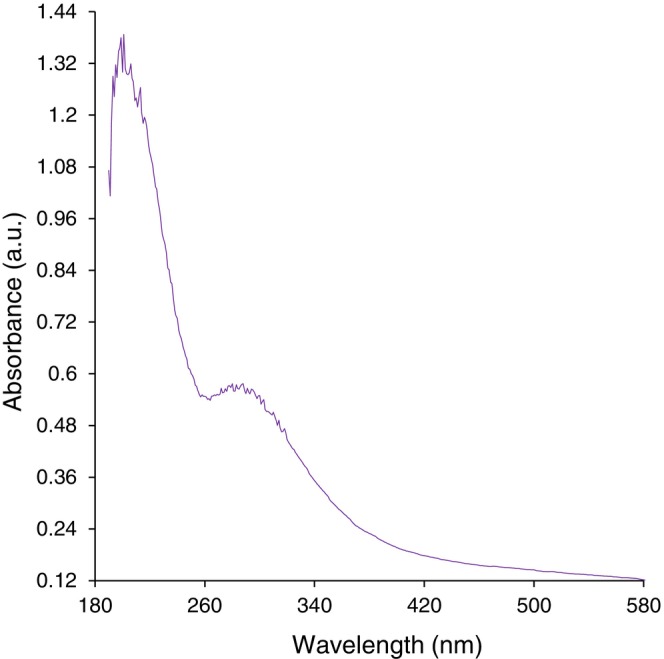
UV–Vis analysis of FeNPs@*Coptis chinensis* in the wavelength of 180–580 nm.

The SPR of the synthetic FeNPs@*Coptis chinensis* was observed by a UV–Vis. The spectrum is exhibited in Figure 2. The band at the wavelength of 294 nm is associated with FeNPs. The nanoparticles have previously been shown to exhibit similar bands (Rezaee et al. 2022). For the FeNPs@*Coptis chinensis* green synthesis, a blue shift is seen in comparison to earlier findings. FeNPs@*Coptis chinensis*'s UV–Vis spectrum may exhibit a blue shift as a result of quantum confinement phenomena. Quantization of energy levels results from electron confinement in very small nanoparticles. As a result, the absorption spectra shift upward toward shorter wavelengths, which look blue‐shifted, due to a greater energy bandgap than in bulk material. Since smaller nanoparticles show stronger quantum confinement effects and, as a result, more significant blue shifts in their UV–Vis absorption spectra, the effect is largely dictated by the shape of the nanoparticles (Kgosiemang et al. 2023; Ahmadi et al. 2021).

**FIGURE 3:**
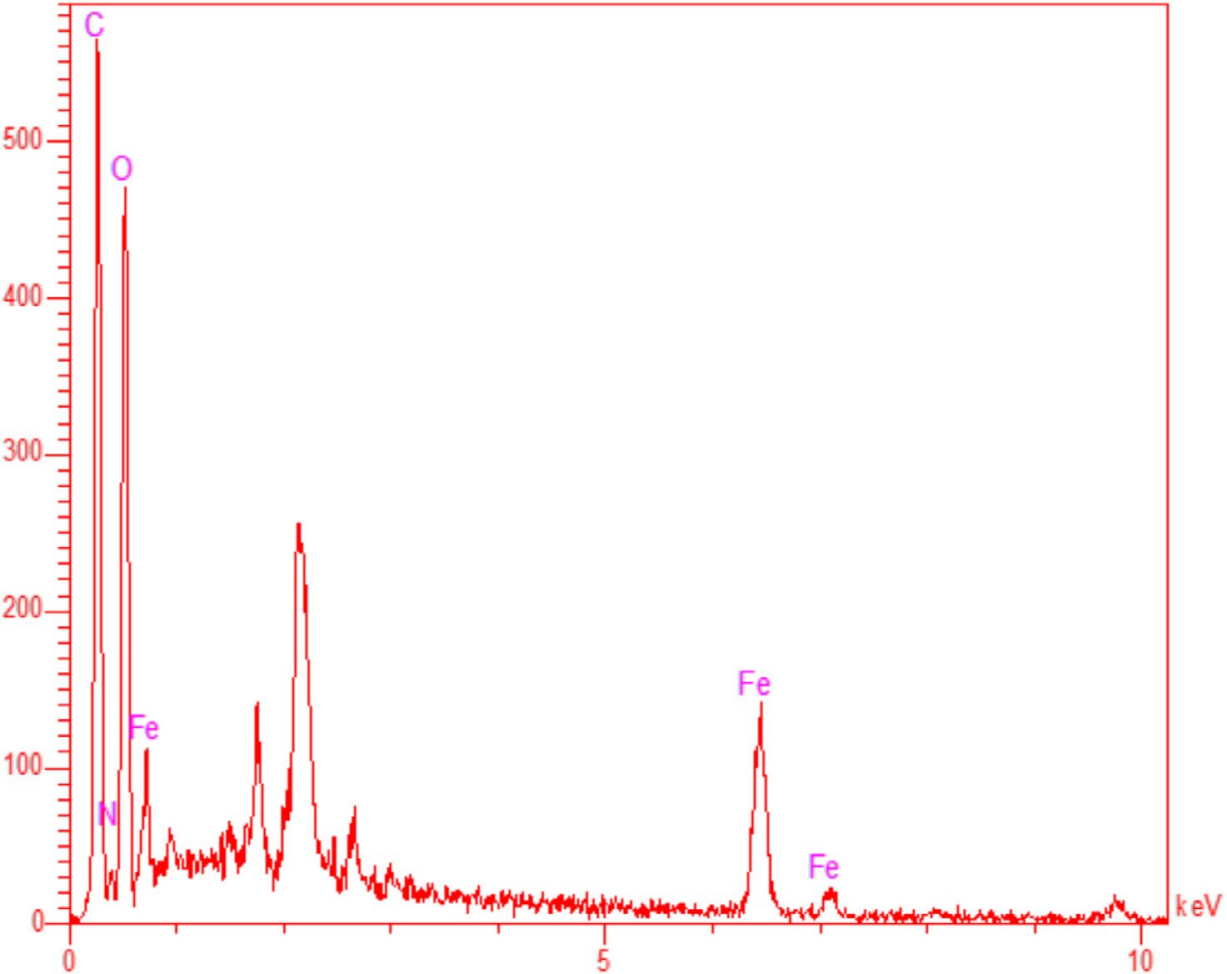
EDX analysis of FeNPs@*Coptis chinensis* in the energy of 0–10 keV.

By using EDX to assess the elemental analysis of Fe NPs, qualitative results were obtained. Figure 3 displays the Fe NPs EDX diagram, which displays the findings of the investigation. The presence of iron was confirmed by the peaks in the obtained data at 6.44 keV for FeKα, 7.13 keV for FeLβ, and 0.71 keV for FeLα. Furthermore, it was shown that the FeNPs included carbon (0.28 keV for CLα) and oxygen (0.52 keV for OLα). Other studies have revealed the iron identification based on the signal (Rezaee et al. 2022; Shi et al. 2021; Kgosiemang et al. 2023; Ahmadi et al. 2021; Namvar et al. 2014; Li and Su 2025). The connection between organic molecules in FeNPs and plant extract has been validated by the oxygen and carbon presence (Ma et al. 2023; Rezaee et al. 2022; Shi et al. 2021; Kgosiemang et al. 2023; Ahmadi et al. 2021).

**FIGURE 4:**
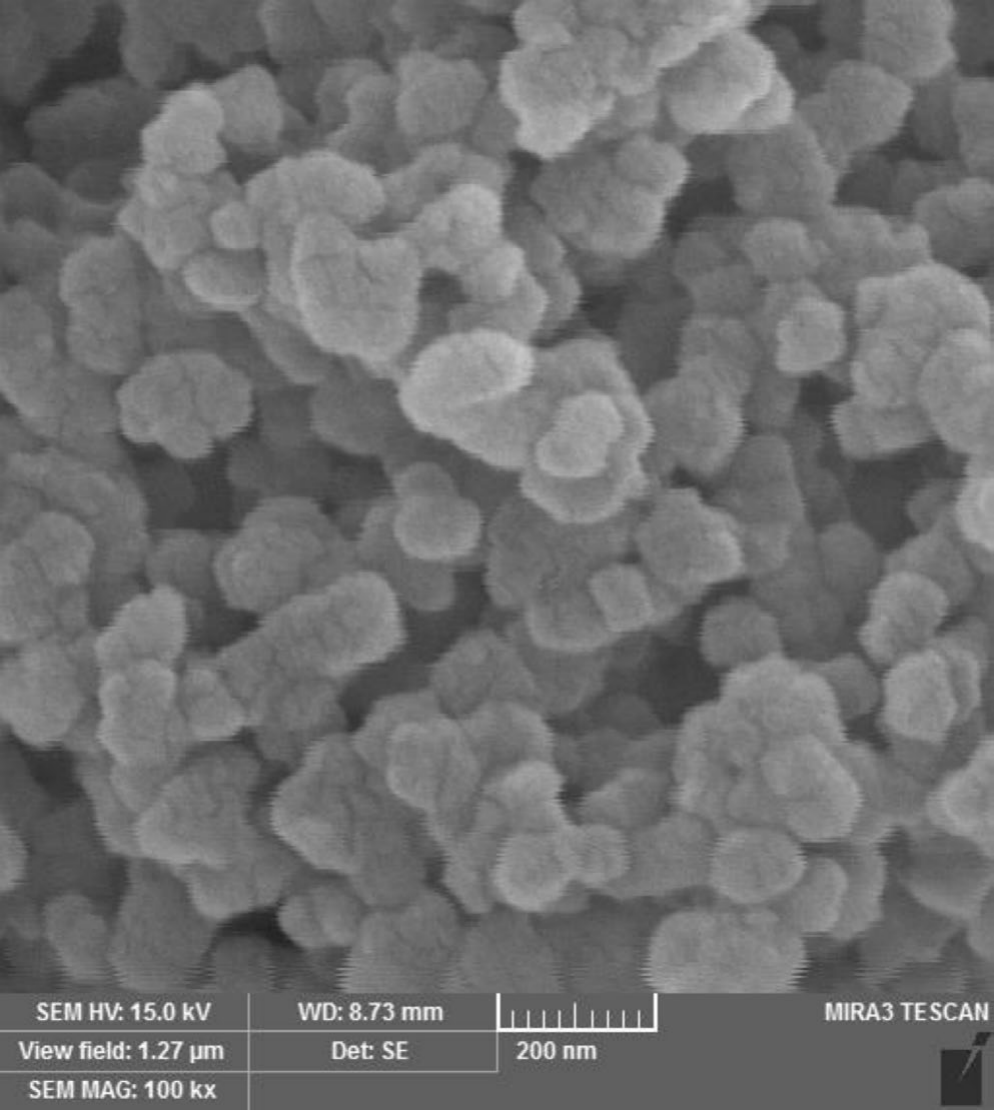
FE‐SEM image of FeNPs@*Coptis chinensis* in the scale bar of 200 nm.

The structure of FeNPs@*Coptis chinensis* was studied by the FE‐SEM imaging method. Figure 4 shows the FeNPs@*Coptis chinensis* morphology. According to the results, the NPs have a spherical shape and an aggregative propensity that is comparable to other metallic nanoparticles that have been reported in the past (Ma et al. 2023; Rezaee et al. 2022; Shi et al. 2021; Kgosiemang et al. 2023). The size distribution from FE‐SEM images shows 5% of nanoparticles in the range sizes of 10–15 nm, 8% in 15–20 nm, 12% in 20–25 nm, 18% in 25–30 nm, 27% in 30–35 nm, 16% in 35–40 nm, 9% in 40–45 nm, and 5% in 45–50 nm for the formulated FeNPs@*Coptis chinensis*. So far, the range size of 20 to 70 nm has been reported for FeNPs (Ma et al. 2023; Rezaee et al. 2022; Shi et al. 2021; Kgosiemang et al. 2023; Ahmadi et al. 2021).

**FIGURE 5:**
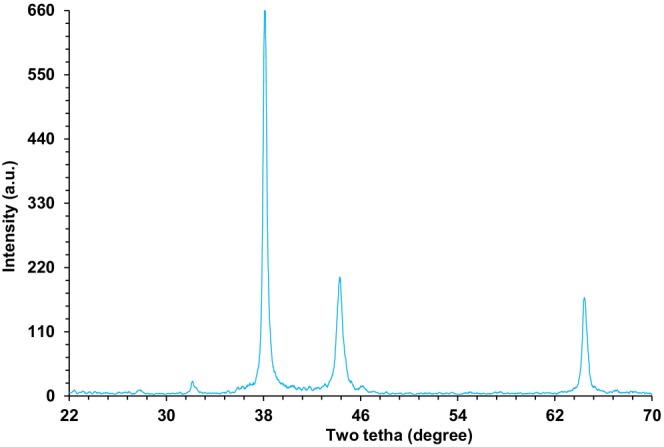
XRD analysis of FeNPs@*Coptis chinensis*.

The crystalline structure and phase of the nanoparticles were determined using X‐ray powder diffraction analysis. The produced nanoparticles have prominent and strong peaks, according to XRD examination, as shown in Figure 5. The ICDD PDF card number 96‐900‐5813 standard database was compared to the collected data. Additionally, the presence of distinct and powerful peaks at a 2*θ* value of 33.2 indicates that the produced nanoparticles are present. With 2*θ* values of 38.3, 44.3, and 64.5, the signals are indexed as (311), (400), and (440) planes. These findings were in line with earlier research (Ma et al. 2023; Rezaee et al. 2022; Shi et al. 2021; Kgosiemang et al. 2023). Using Scherrer's formula, the crystalline grain size of the biogenic Fe_2_O_3_ was found to be as follows: *L* = *Kλ*/*β* cos *θ*, where K is Scherrer's constant (0.94), *ω* is the X‐ray wavelength (*λ* = 1.5406 Å for copper), and *β* is the full width at the half‐maximum (FWHM) of the strongest peak at 2*θ* ^2^ of 33.52°, which was determined to be 0.1824. The FeNPs@*Coptis chinensis* were found to have a crystalline size of 42.37 nm.

Inflammation, which includes immune system cell activation and the production of inflammatory protein cytokines, is the immune system's response to tissue damage. Furthermore, inflammation is a crucial response to several stimuli, such as toxins, hypoxia, infections, microbes, tissue damage, immunological reactions, and physical and chemical harm (Chen et al. 2018). As a result, foreign materials are eliminated, and tissue structure and physiological function are restored. However, inflammation starts as a protective mechanism and can result in lupus nephritis and other inflammatory conditions if this complex process is not managed (Calder et al. 2009). In this way, macrophages start eating the damaged tissues when inflammation happens. One of the numerous cancers that are gradually caused by this process is liver carcinoma. Most interleukins decrease tissue inflammation while also strengthening the host's defenses. By removing the inflammatory agent, destroying the damaged tissue parts, and finally mending the tissue, they prevent the inflammatory diseases such as periodontitis (Cardoso et al. 2018). An inflammatory response that damages the tooth's connective tissue and bone is called periodontitis. Gingivitis, an inflammation of the gums brought on by bacterial plaque accumulation on the tooth surface, develops into periodontitis if treatment is not received. The loss of dental tissues ultimately leads to tooth loss. Tooth loss may result from untreated periodontitis. Additionally, there is an increased heart attack, stroke, and other health problems. Bacterial plaque is the most common cause of periodontitis; it is a sticky, white film that develops on the surface of teeth. Plaque can solidify and become tartar if it is not removed from the teeth (Calder et al. 2009; Cardoso et al. 2018). This illustrates how crucial it is to get your teeth scaled by a dentist and to see him regularly. The utilization of natural substances derived from metallic nanoparticles has aided in the development of innovative anti‐inflammatory supplements with reduced toxicity and substantial remedial effects in the treatment of disorders. The NPs have recently been used to treat inflammation and wounds (Hebeish et al. 2014). Among the response features that can be linked to the high anti‐inflammatory efficacy of the nanoparticles are increased vascular permeability, white blood cell activation, prostaglandin inflammatory release, and the immune system cytokines, such as interleukins and granulocyte factor stimulator (Alsareii et al. 2022).

**FIGURE 6:**
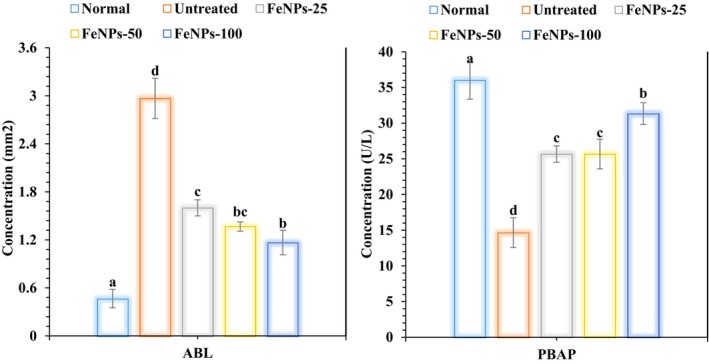
The effects of FeNPs ABL and PBAP in groups with periodontitis (*n* = 10, *p* < 0.05).

FeNPs administration significantly increased plasma bone ALP levels (measured in U/L) and decreased alveolar bone loss (measured in mm^2^) in a study on experimental periodontitis as compared to the untreated group. The amounts of plasma bone alkaline phosphatase and alveolar bone loss varied statistically significantly (*p* < 0.05) among dosages of 25, 50, and 100 μg/kg (Figure 6). ABL and PBAP serve as key markers in assessing bone regeneration outcomes, particularly in periodontitis and osteoporosis models where bone resorption is prominent. ABL quantifies linear or volumetric loss of alveolar bone via micro‐CT or histomorphometry, while elevated PBAP indicates increased bone turnover and osteoblast activity, often correlating with regeneration potential (Sun and Yu 2024).

**FIGURE 7:**
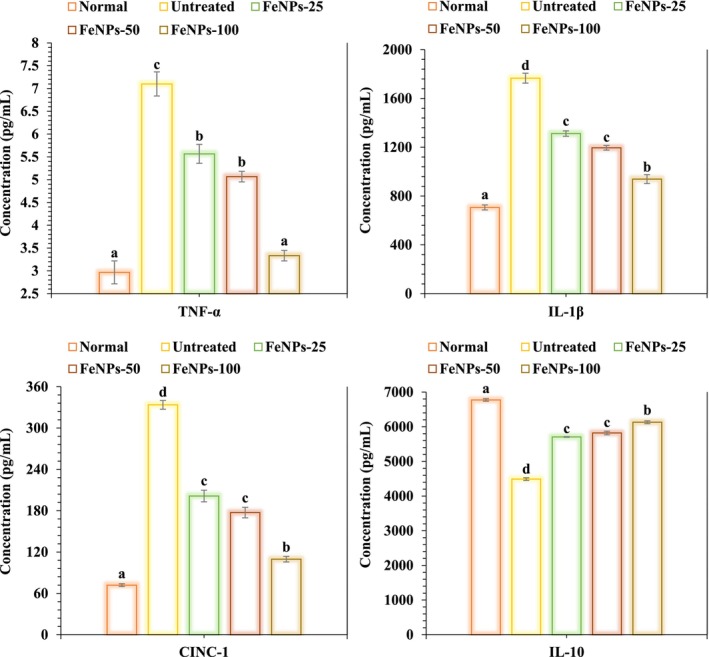
The effects of FeNPs on inflammatory cytokines in gingival tissues in groups with periodontitis (*n* = 10, *p* < 0.05).

Researchers found that periodontal rats treated with FeNPs at dosages of 25, 50, and 100 μg/kg had higher levels of anti‐inflammatory cytokines (pg/mL), like IL10, and lower levels of pro‐inflammatory cytokines (pg/mL), like CINC‐1, TNF‐α, and IL1‐β. There is a significant difference (*p* < 0.05) in the inflammatory cytokine concentrations, including CINC‐1, TNF‐α, and IL‐10, between dosages of 25 and 50 μg/kg and the 100 μg/kg dosage (Figure 7).

**FIGURE 8:**
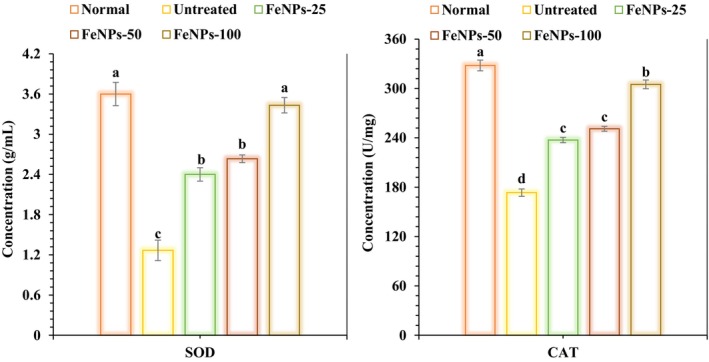
The effects of FeNPs on periodontitis groups' gingival tissues, namely on CAT and SOD (*n* = 10, *p* < 0.05).

FeNPs increased catalase (U/mg) and superoxide dismutase (g/mL) levels in experimental periodontitis compared to the untreated group. There is a significant difference (*p* < 0.05) in the concentrations of catalase and superoxide dismutase between the 25 and 50 μg/kg dosages and 100 μg/kg dosage (Figure 8).

**FIGURE 9:**
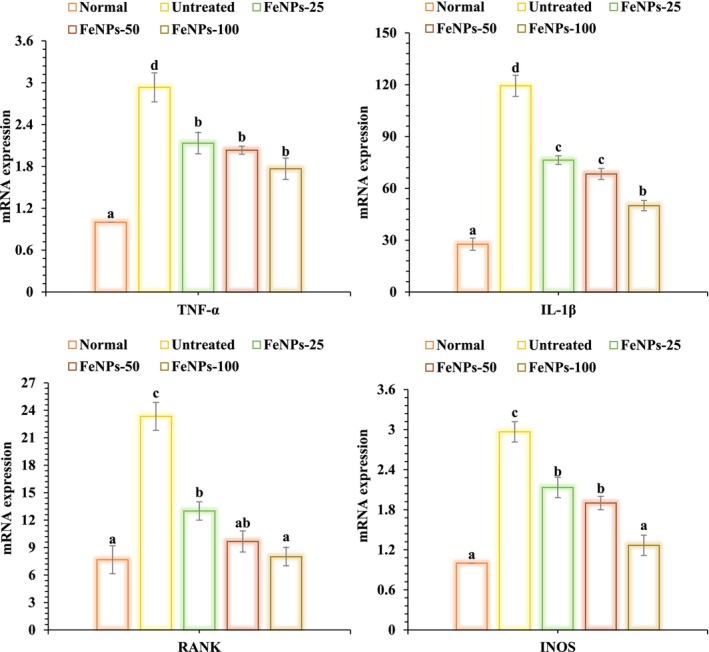
The effects of FeNPs on periodontitis groups' gene mRNA expression.

The mRNA expression of the RANK, TNF‐α, and IL1‐β genes decreased when rats with periodontitis were given FeNPs at doses of 25, 50, and 100 μg/kg. There is a substantial difference (*p* < 0.05) in TNF‐α levels and the iNOS gene's mRNA expression between the 25 and 50 μg/kg dosages and the 100 μg/kg dosage (Figure 9).

FeNPs modulate TNF‐α and IL‐1β primarily by suppressing their production in immune cells like microglia and monocytes during inflammatory responses, such as those triggered by lipopolysaccharide (Wu et al. 2013). FeNPs inhibit IL‐1β secretion via the secretory lysosomal pathway in murine microglial cells, reducing pro‐inflammatory cytokine release without affecting cell viability. In primary human monocytes, FeNPs dampen LPS‐induced inflammation, lowering TNF‐α and IL‐1β levels through altered signaling pathways. This anti‐inflammatory effect contrasts with some reports where FeNPs enhance IL‐1β and TNF‐α in macrophages, depending on FeNPs' properties like size and coating (Dahri et al. 2025). In infection models, FeNPs' cytokine modulation supports antimicrobial therapy by curbing excessive inflammation while enhancing antibiotic efficacy against pathogens like *Streptococcus pneumoniae* . Surface modifications, such as carboxymethyl dextran, further influence complement activation and immune modulation, potentially amplifying adaptive responses. Research remains mostly preclinical, with effects varying by cell type and exposure conditions (Ansari et al. 2024).

Several green‐synthesized nanoparticles have been tested in inflammation and periodontitis models, including plant/propolis‐mediated metal nanoparticles and propolis‐loaded lipid nanocarriers. Most data are in vitro on periodontal pathogens or in vivo in ligature‐induced rat periodontitis (Barik et al. 2024). Plant‐ or herbal‐extract mediated silver nanoparticles (AgNPs) synthesized using neem ( *Azadirachta indica* ), amla ( *Emblica officinalis* ), and other medicinal plants show strong antibacterial and antibiofilm activity against key periodontal pathogens such as *Porphyromonas gingivalis* and other plaque‐associated bacteria, along with reported antioxidant and anti‐inflammatory potential. These systems use plant phytochemicals as reducers and stabilizers, making the synthesis less toxic and more biocompatible than conventional chemical routes (Anjum et al. 2016). Propolis‐mediated AgNPs, including those from Iranian green propolis, significantly inhibit *P. gingivalis* biofilm formation in vitro and are proposed as adjuncts to periodontal therapy. Propolis provides phenolic and flavonoid compounds that contribute additional antimicrobial and anti‐inflammatory effects beyond the metallic core (Hedayatipanah et al. 2024). Green propolis‐loaded lipid nanoparticles (GPlnp) have been evaluated in an ovariectomized rat model with zoledronate and ligature‐induced experimental periodontitis, where local irrigation with GPlnp was used as an adjunct to scaling and root planing. In this model, GPlnp improved clinical, histologic, and immunohistochemical markers, reducing local inflammatory response and expression of TNFα and IL‐1β, and helping preserve periodontal tissues in comparison to control therapies (Silveira et al. 2024). These lipid nanoparticles are described as biocompatible nanocarriers that enhance the delivery of green propolis constituents with anti‐inflammatory, antioxidant, antimicrobial, and tissue‐repair–stimulating actions, aiming to modulate inflammation and bone loss in periodontitis (Silveira et al. 2024).

Green‐synthesized metallic nanoparticles from plant sources show combined actions relevant to periodontal inflammation: Antibacterial and antibiofilm activity against subgingival pathogens, scavenging of reactive oxygen species, and downregulation of pro‐inflammatory mediators in cell and animal models. In ligature‐induced periodontitis models, nanoformulations that modulate oxidative stress and inflammatory signaling can reduce the distance between the cementoenamel junction and alveolar bone crest and improve bone volume parameters, indicating protection against inflammatory bone resorption (Navya et al. 2023). Reviews on nanoparticles in periodontitis therapy highlight that green‐synthesized NPs, when properly formulated, can both control biofilms and modulate host inflammatory responses while supporting periodontal regeneration, but most candidates remain at preclinical stages (Wang et al. 2024).

## 4 Conclusion

We have successfully created a new, environmentally friendly process for creating FeNPs by employing an extract from *C. chinensis* as a bio‐reductant. The production of highly crystalline, semi‐spherical Fe NPs with a consistent size distribution is made possible by this method, which is essential for their possible uses in biomedical devices, sensing, and catalysis. Flavonoids and phenolic acids are two examples of the phytomolecules found in plants that not only aid in the reduction of iron ions but also serve as organic capping agents by attaching to the surface of the Fe NPs and stopping their aggregation. This was confirmed using FT‐IR spectroscopic analysis, which demonstrated the presence of particular functional groups on the Fe NPs surface. The *C. chinensis* extract‐capped Fe NPs' biocompatibility and biodegradability make them a desirable substitute for conventional synthetic processes, which frequently call for capping ligands and hazardous reducing agents. Fe NPs decreased the levels of alveolar bone loss, PBAP, antioxidant enzymes, inflammatory cytokines, and the mRNA expression of the iNOS, RANK, IL1‐β, and TNF‐α genes in periodontal rats. Due to the *C. chinensis* components (berberine, flavonoids) having significant anti‐inflammatory effects against the pro‐inflammatory cytokines, it seems that the synergistic effects of *C. chinensis* extract and iron reaction in green formulation make higher anti‐inflammatory effects in a rat model of periodontitis. FeNPs green mediated by *C. chinensis* extract must undergo rigorous toxicology studies, including long‐term biocompatibility, biodistribution, and genotoxicity assessments in larger animal models before human trials, as current data remain limited to in vitro and rodent ligature‐induced periodontitis. Future designs should integrate antimicrobial action with host modulation, such as combining FeNPs with anti‐inflammatory payloads or cell membrane coatings for immune evasion and enhanced biofilm penetration in complex periodontitis microbiomes. Hybrid systems, like upconversion FeNPs for light‐activated photodynamic therapy or enzyme‐FeNPs conjugates, could target deep subgingival pockets while promoting regeneration via ROS regulation and osteogenic signaling in alveolar bone. Testing in advanced models, including obese/diabetic rats or patient‐derived organoids, will better mimic human disease heterogeneity. Regulatory pathways for dental nanomaterials, such as FDA approval for adjunctive periodontal gels or irrigants, demand evidence of superiority over scaling/root planing plus antibiotics.

## Author Contributions

Jialing Wu, Xinjie Gao, and Ruiqi Li have the same role in conceptualization, investigation, acquisition, formal analysis, data curation, supervision, project administration, methodology, writing – original draft, and writing – review and editing. The authors confirm that no paper mill or artificial intelligence was used.

## Funding

This work was supported by the Scientific Research Cultivation Program of Stomatological Hospital, Southern Medical University (Grants PY2022031 and PY2022030).

## Ethics Statement

The experiments were performed according to the ethical guidelines of the International Association for the Studies of in vitro, in vivo, and humans confirmed by Southern Medical University, S366 Jiangnan Boulevard, Haizhu District, Guangzhou, Guangdong, 510280, China.

## Consent

The plant was separately collected from the local market and identified at the Botany Department, Southern Medical University, S366 Jiangnan Boulevard, Haizhu District, Guangzhou, Guangdong, 510280, China.

## Conflicts of Interest

The authors declare no conflicts of interest.

## Data Availability

Data is available on request from the corresponding author.
